# Le traitement chirurgical des cals vicieux des deux os de l'avant-bras: à propos d'une série de 11 cas

**DOI:** 10.11604/pamj.2013.14.41.2300

**Published:** 2013-01-29

**Authors:** Mohammed Elidrissi, Atif Mechchat, Hatim Abid, Mohammed Shimi, Abdelhalim Elibrahimi, Abdelmajid Elmrini

**Affiliations:** 1 Service de chirurgie ostéoarticulaire B4, CHU Hassan II, Fès, Maroc

**Keywords:** Cals vicieux, avant-bras, amplitudes préoperatoires, amplitudes postperatoires, radius, ostéotomie, malunion, forearm, preoperative amplitudes, postoperative amplitudes, radius, osteotomy

## Abstract

Le but de cette étude est de présenter l'expérience du service de chirurgie ostéoarticulaire B4, de CHU Hassan II de Fès Maroc, dans la prise en charge chirurgicale des cals vicieux des deux os de l'avant-bras. C'est une étude rétrospective étalée entre janvier 2008 et décembre 2011 incluant onze cas de cal vicieux de l'avant-bras chez des adultes, colligés au service de chirurgie ostéoarticulaire B4 du CHU Hassan II de Fès. Pour chaque patient nous avons étudié: Sur le plan clinique: la profession, la nature du traumatisme initial, le traitement initial, l'amplitude de pronosupination. Sur le plan radiologique: l'aspect radiologique du cal. La limitation de la pronosupination était le principal motif de consultation, trois patients ont consulté pour la déformation. Le recul moyen est de 18 mois, avec des extrêmes de 5 mois et 48 mois. La prise en charge chirurgicale avait permis d'améliorer de façon variable chez tous les patients les amplitudes de pronosupination de 58° en moyenne. Les résultats étaient bons chez 5 patients, moyens chez 5 et mauvais chez un patient. Le traitement conservateur des fractures des deux os de l'avant-bras est incriminé dans la genèse des cals vicieux de l'avant-bras. Le traitement chirurgical de ceux-ci fait appel à une ostéotomie de correction. Dans notre étude nous avons montré l'intérêt du rétablissement des axes du radius et de l'ulna pour la restauration de la fonction de pronosupination, grâce à cette ostéotomie.

## Introduction

La survenue d'une fracture au niveau de l'un ou des deux os de l'avant-bras met en jeu la précision du positionnement de la main dans l'espace, en raison de la perturbation des mouvements de pronosupination. C'est la raison pour laquelle la conviction des chirurgiens orthopédistes est allée vers le traitement sanglant de toute fracture des deux os de l'avant-bras. Cette attitude explique la rareté de cals vicieuse des deux os de l'avant-bras dans les pays industrialisés, situation devenue l'apanage des fractures traitées orthopédiquement. Par contre elle pose encore un problème thérapeutique et fonctionnel dans les pays en voie de développement. L'objectif de notre étude est de présenter notre expérience dans la prise en charge des cal vicieux de l'avant-bras.

## Méthodes

Nous présentons une étude rétrospective étalée entre janvier 2008 et décembre 2011 incluant onze cas de cal vicieux de l'avant-bras chez des adultes, colligés au service de chirurgie ostéoarticulaire B4 du CHU Hassan II de Fès.

**Critères d'inclusion:** tous les patients ayant un cal vicieux de l'avant-bras.

**Critères d'exclusion:** toute fracture fraiche, ainsi que les pseudarthroses.

La survenue d'une fracture au niveau de l'un ou des deux os de l'avant-bras met en jeu la précision du positionnement de la main dans l'espace, en raison de la perturbation des mouvements de pronosupination. C'est la raison pour laquelle la conviction des chirurgiens orthopédistes est allée vers le traitement sanglant de toute fracture des deux os de l'avant-bras. Cette attitude explique la rareté de cals vicieuse des deux os de l'avant-bras dans les pays industrialisés, situation devenue l'apanage des fractures traitées orthopédiquement. Par contre elle pose encore un problème thérapeutique et fonctionnel dans les pays en voie de développement. L'objectif de notre étude est de présenter notre expérience dans la prise en charge des cal vicieux de l'avant-bras.

Pour chaque patient nous avons étudié: sur le plan clinique: la profession, la nature du traumatisme initial, le traitement initial, l'amplitude de pronosupination; sur le plan radiologique: l'aspect radiologique du cal.

## Résultats

Parmi ces onze patients, 7 étaient des hommes, et 4 femmes. La moyenne d'âge est de 31ans, avec des extrêmes d'âge de 17 ans et 48 ans. Tous nos patients étaient des travailleurs manuels, le côté atteint était droit chez 80% d'entre eux. Les patients ont consulté à un délai qui varie entre deux mois et 18 mois, le traumatisme initial était une simple chute de hauteur chez la majorité des patients (8 cas), un accident de voie publique chez 2 patients, et une agression chez 1 patient. Sept (7) d'entre ces patients ont subit un traitement traditionnel, et 4 traitement orthopédique. Sur le plan clinique la limitation de la pronosupination était le principal motif de consultation (8 patients), trois patients ont consulté pour la déformation. Les amplitudes de pronosupination sont représentées sur le Tableau 1.

**Tableau 1:** Les amplitudes préoperatoires de pronosupination

Amplitudes de pronosupination (degrés)	120	90	85	70	60
Nombre patient	2	2	3	3	1

La majorité de nos patients avait une flexion et extension conservée (7 patients), deux patients avaient une raideur du coude en flexion à 90°, et deux avaient une limitation de l'extension également.

Sur le plan radiologique, le cal intéressait les deux os de l'avant-bras en médiodiaphysaire chez sept patients, deux patients avait une fracture de Montegea négligée, un patient avait un cal vicieux du quart distal du radius avec bonne consolidation du cubitus, et un patient présentait un cal vicieux du radius avec une pseudarthrose de cubitus. Le cal vicieux était angulaire en flexion chez 10 de nos patients avec un angle qui varie entre 11° et 40°, et seulement un patient présentait un cal vicieux rotatoire du quart distal du radius.

**Figure 1:**
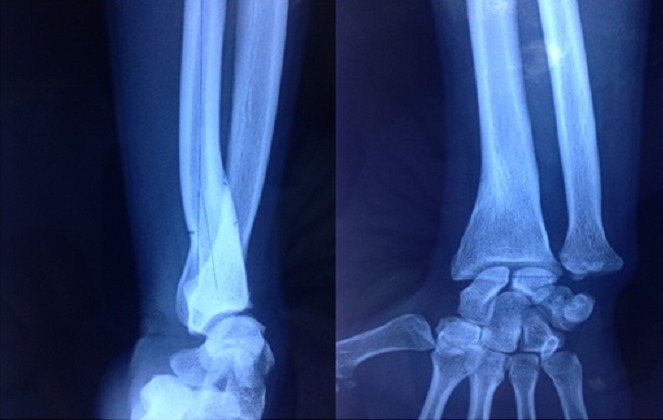
Cal vicieux angulaire du radius droit

**Figure 2:**
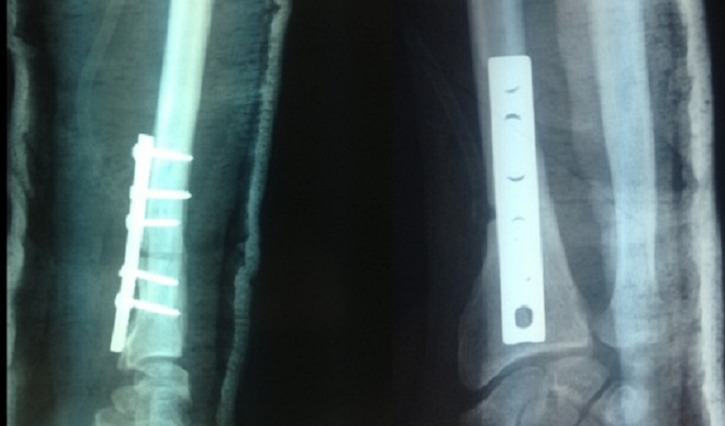
Contrôle radiologique après ostéotomie

**Figure 3:**
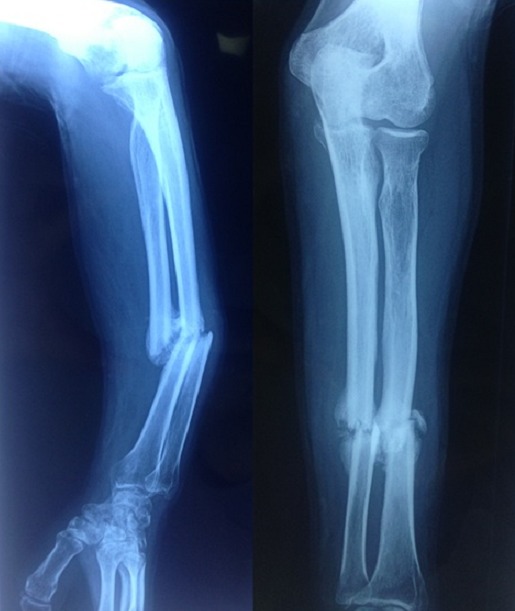
Cal vicieux des deux os de l'avant-bras gauche

**Figure 4:**
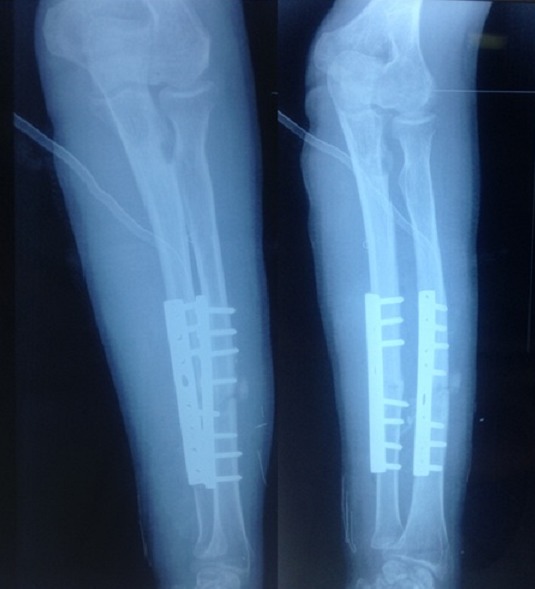
Contrôle postopératoire après ostéotomie

La prise en charge de ces onze patients était chirurgicale, par abord direct du cal on a réalisé une ostéotomie avec fixation par une plaque vissée DCP et vissage par des vis corticales 3,5 des deux os de l'avant-bras chez sept patients, du cubitus chez les deux patients qui présentent une fracture de Montegea négligée, la réduction de la luxation de la tête radiale a été obtenue après rétablissement de l'axe de l'ulna, et une ostéotomie de réorientation du quart distal du radius avec fixation par une plaque vissée distale du radius, une ostéotomie du radius et une décortication greffe a été faite chez le patient qui présente la pseudarthrose du cubitus associée (Figure 1, Figure 2, Figure 3, Figure 4).

Le recul moyen est de 18 mois, avec des extrêmes de 5 mois et 48 mois, un seul patient avait présenté un sepsis superficiel en postopératoire, bien tarit par une antibiothérapie adaptée.

Nous avons évalué les résultats en fonction des amplitudes de pronosupination gagnée en postopératoire, les patient ont étaient classés en trois groupe:

- Bon résultats: la pronosupination entre 160° et 180°
- Bon résultats: la pronosupination entre 160° et 180°
- Résultat moyen: la pronosupination entre 90° et 160°
- Mauvais résultat: la pronosupination inférieure à 90°

Le Tableau 2 résume les amplitudes de pronosupination postopératoire.

**Tableau 2:** Les amplitudes postperatoires de pronosupination

Amplitudes de pronosupination (en degrés)	175	165	150	140	80
Nombre patient	5	3	1	1	1

## Discussion

Le but du traitement des fractures des deux os de l'avant-bras est la consolidation ainsi que la restauration de la fonction de pronosupination. La restauration de cette fonction dépend de la préservation de la longueur du squelette et de l′alignement axial et rotationnel [1]. Les cals vicieux post-traumatiques de l'avant-bras entraine une limitation des amplitudes de pronosupination, des douleurs de l'articulation radio-ulnaire inférieure, et des problèmes esthétiques [2].

Dans notre série, nous avons noté la fréquence des cals vicieux de l'avant-bras chez le sujet jeune de sexe masculin, ce qui rejoint les données de la littérature [3, 4]. Cette prédominance est expliquée par l'activité relativement élevée de cette [5, 6]. La chute de sa hauteur est le mécanisme le plus retrouvé [7].

Il a été démontré que le traitement orthopédique des fractures de l'avant-bras et plus pourvoyeur de cal vicieux [1]. C'est pour cela que actuellement la majorité des fractures de l'avant-bras sont traitées chirurgicalement ceci permet de minimiser le risque de défaut d'alignement ainsi que de diminuer la perte en pronosupination [1]. Un traitement chirurgical mal conduit peut également évoluer vers le cal vicieux [6]. Dans notre série ce sont des patients qui ont subi un traitement traditionnel par des moyens d'immobilisation de fortune faits par des tradipraticiens.

Nous avons également noté la prédominance des cals vicieux angulaires, par rapport aux cals vicieux rotatoires ceci rejoint les résultats de littérature [3, 4, 6]. Dans une étude expérimentale, Matthews et al. avaient montré le retentissement des cals vicieux angulaires sur la fonction de pronosupination de l'avant-bras. Ce retentissement est beaucoup plus net que l'angulation dépasse les dix [8], cependant le cal vicieux rotatoire a été retrouvé chez un patient. Ces défauts de rotation sont considérés comme plus graves car ils entrainent une limitation de la pronosupination à une amplitude qui égale au degré du cal vicieux rotatoire.

L'ostéotomie correctrice est le traitement de choix des cals vicieux de l'avant-bras. Les indications de cette ostéotomie sont: les troubles de la pronosupination, une instabilité de l'articulation radionulnaire distal, et une déformation inacceptable sur le plan esthétique [9].

## Conclusion

Les deux os de l'avant-bras, avec les articulations radio-ulnaires supérieures et inférieures, ont une disposition en cadre, ce qui leur permet d'assurer leur fonction principale: la pronosupination. Dans notre série nous avons montré l'intérêt du rétablissement des axes du radius et de l'ulna pour la restauration de la fonction de pronosupination. Et ceci grâce à une ostéotomie de correction avec la fixation par un moyen d'ostéosynthèse stable, et une rééducation bien suivie dans un centre spécialisé.

## References

[1] Evans EM (1951). Fractures Of The Radius and Ulna. J Bone Joint Surg Br..

[2] Nagy L, Jankauskas L, Dumont CE (2008). Correction of forearm malunion guided by the preoperative complaint. Clin Orthop Relat Res..

[3] Dupuis JF, Furno P (1978). Cals vicieux de l'avant-bras. Actualités de chirurgie orthopédique de l'hôpital Raymond-Poincaré et de l'institut de recherches orthopédiques..

[4] Labe A (1978). Pseudarthrose et cals vicieux après fractures diaphysaires des deux os de l'avant-bras chez l'adulte.

[5] Singer BR, McLauchlan GJ, Robinson CM, Christie J (1998). Epidemiology of fractures in 15,000 adults: the influence of age and gender. J Bone Joint Surg Br..

[6] Bousso A (2007). Cals vicieux diaphysaires des deux os de l'avant-bras chez l'adulte - A propos de dix observations. Chirurgie de la main..

[7] McQueen MM, Wakefield A (2008). Distal radial osteotomy for malunion using non-bridging external fixation: good results in 23 patients. Acta Orthop..

[8] Matthews LS, Kaufer H, Garver DF, Sonstegard DA (1982). The effect on supination-pronation of angular malalignment of fractures of both bones of the forearm. J Bone Joint Surg Am..

[9] Trousdale RT, Linscheid RL (1995). Operative treatment of malunited fractures of the forearm. J Bone Joint Surg Am..

